# Late fetal demise, a risk factor for post-traumatic stress disorder

**DOI:** 10.1038/s41598-022-16683-5

**Published:** 2022-07-20

**Authors:** Lucile Abiola, Guillaume Legendre, Andrew Spiers, Elsa Parot-Schinkel, Jean-François Hamel, Philippe Duverger, Pierre-Emmanuel Bouet, Philippe Descamps, Caroline Quelen, Philippe Gillard, Elise Riquin

**Affiliations:** 1 Department of Obstetrics and Gynecology, Angers University Hospital, Angers, France; 2 CESP-Inserm, U1018, Team 7, Genre, Sexual and Reproductive Health, Paris-Sud University, Paris-Saclay University, UVSQ, Inserm, Villejuif, France; 3 CESP-Inserm, U1018, Research in Ethics and Epistemology (R2E), Paris-Sud University, Paris-Saclay University, Inserm, Paris, France; 4 Department of Clinical Research and Innovation, Angers University Hospital, 4 Rue Larrey, 49100 Angers, France; 5 Department of Child Psychiatry, Angers University Hospital, Angers, France; 6 Department of Psychology Laboratoire LPPLEA4638, Angers University, Angers, France; 7 Unité Micovasc, UMR CNRS 6015-INSERM, 1083 Angers, France

**Keywords:** Medical research, Health care

## Abstract

Late-term fetal demise including fetal death in utero, late miscarriage and late termination of pregnancy are relatively frequent occurrences. Post-traumatic stress disorder (PTSD) is a pathology that finds its roots in exposure to a life-threatening event or an event related to death. Exposure to fetal death during a late-term fetal demise is, therefore, a situation at risk of trauma. The objective of this study was to assess the prevalence of PTSD symptoms in the short term among patients faced with late fetal demise, and to identify potential risk factors. All women were assessed at 15 days, one month, and three months after late fetal demise using the Impact of Event Scale-Revised (IES-R) and the Peritraumatic Dissociative Experiences Questionnaire (PDEQ). At 15 days, 44.2% of patients presented a pathological score on the IES-R (≥ 33). At one month and three months, this figure was 34.1% and 9.1% respectively. Factor associated with PTSD symptoms were: peritraumatic dissociation (p = 0.014), seeing the fetus during hospitalization (p = 0.035), holding the fetus in one’s arms (p = 0.046), and the organization of a funeral service (p = 0.025). Patients experiencing late fetal demise are at significant risk of trauma. Care providers should remain vigilant to identify high-risk situations to offer appropriate care.

Clinical trials registration number: NCT03433989.

## Introduction

Late fetal demise, defined in our study as per the French guidelines, is^1^:

- Late Miscarriage (LM): spontaneous and unplanned evacuation of an intrauterine pregnancy after 14 WG and before 22 WG (weeks of gestation). The expulsion of a fetus from the womb before it is able to survive independently.
- Fetal death in utero (FDIU) after 14 WG: spontaneous cessation of fetal heart activity at or after 14 WG. The diagnosis of fetal death is made in the absence of fetal movements perceived by the mother or during an ultrasound examination. Delivery is more often than not induced as the FDIU is responsible for co-morbidities in the mother if it is maintained too long (risk of coagulation disorders in particular).
- Late termination of pregnancy (LTP), which is authorized in France regardless of term, is a relatively frequent occurrence. There is no clear consensus on the definition of “late termination of pregnancy” although in current scientific literature, the term is frequently used to describe medically assisted interruption of pregnancy during the 2nd and 3rd trimesters. The French law allows LTP in the event of a serious maternal illness that endangers the life of the mother or in the event of an incurable fetal abnormality or serious illness.

The prevalence of late fetal demise is approximately 3 to 4% of all pregnancies^2^.

Late fetal demise is associated with particularly severe grief and a complex mourning process^3,4^. The loss of pregnancy and fetal death, through LM, FDIU or LTP is recognized as a traumatic life event^3–5^. After a traumatic event, psychiatric symptoms can occur. According to the DSM-5, acute stress disorder (ASD) is defined by the presence of nine symptoms divided into five categories: intrusion symptoms, alteration in arousal and reactivity, negative alterations in cognition and mood, dissociative symptoms (such as amnesia or alteration in the perception of the reality) and avoidance symptoms^6^. ASD occurs from three days to one month after exposure to a traumatic event. If symptoms persist for longer than one month or if they arise after a period of one month, then a diagnosis of a “post traumatic stress disorder” (PTSD) can be put forward. According to the DSM-5, symptoms include the existence of distress or symptoms of intrusion, avoidance, negative alterations in cognitions and mood and alterations in arousal and reactivity^7^.

After a delivery leading to the birth of a live new born, PTSD prevalence is about 3%^8,9^. The risk factors associated with the development of PTSD after childbirth are: maternal psychopathological antecedents and exposure to a traumatic event, medical complications at the time of delivery, but also poor quality of interaction with caregivers and doctors at the time of delivery and later the lack of social support (especially from spouses) during the postpartum period^8,10^.

The prevalence of PTSD symptoms among patients having experienced a late fetal demise ranges between 0.6 and 39%^4^. This psychiatric pathology adds further difficulty to an already complex mourning process.

With regard to perinatal bereavement, the majority of studies focus on the situation after a LTP, or on the long term wellbeing of patients^11–13^. These studies show an 18 to 39% prevalence of PTSD symptoms in the aftermath of late fetal demise. Others studies show that PTSD can have long term consequences if effective treatment is not provided after an LTP^14^. Factors that compound the risk of PTSD have also been identified, such as holding or seeing one's dead baby^15,16^. These studies focus on mothers at a given time but, to our best knowledge, no study has focused on the evolution of symptoms through time by evaluating women several times after late fetal demise.

Another risk factor for developing post-traumatic stress symptoms, that has been described in literature, is peritraumatic dissociation at the time of the event is defined as a disruption, interruption, and/or discontinuity of normal, subjective integration of behaviour, memory, identity, consciousness, emotion, perception, body representation, and motor control^17,18^.

The objective of this study was twofold: firstly to assess the prevalence of PTSD symptoms in the short term following late fetal demise and, secondly, to identify the risk factors associated with PTSD.

## Materials and methods

We conducted a prospective and descriptive study between March 2018 and December 2019. All patients who experienced a late fetal demise in the maternity ward of the Angers University Hospital were eligible for inclusion. All patients were assessed at 15 days, one month and three months after late fetal demise. All data collected was anonymized. The study received approval from the Research Ethics Board, South-East 1-Personal Protection Committee (CPP) number 2017-102588-45.

The study methodology was performed in accordance with the Declaration of Helsinki, and in compliance with regulations, legislation and guidelines for human respect.

In total, 156 patients were eligible during the inclusion period and 109 patients agreed to participate in the study. Patients were recruited according to the following criteria:

- Inclusion criteria: patients hospitalized, at the Angers University Hospital for LM, FDIU and LTP and who agreed to participate in the study. It was decided that only patients beyond 14WG should be included because, the termination of pregnancy at this stage can no longer be voluntary and can only be done for medical purposes. Furthermore, beyond 14 WG, the fetus can be seen by the parents and can be acknowledged to the register of births according to French law. For LTP and FDIU, patients were included from 14 WG to term. For LM, patients were eligible for inclusion in the study from 14 to 22 WG, in accordance with the definition of LM.
- Exclusion criteria: patients under the age of 18, non-French speaking patient, patients already partaking in another study, patients with a known psychiatric disorder at the time of late fetal demise (according to patients’ declaration regarding their background).

Pre-selection of patients was carried out daily by the investigator from the register of births and medical records, informed consent was then obtained and written information provided.

After informed consent had been given, the demographic characteristics of the patient, the history of the pregnancy, previous hospitalization and the legislative and administrative choices concerning the fetus and corpse (organization of a funeral service or handing-over of the body to the hospital, declaration to civil authorities, attribution of a first name) were all collected from the patients' medical files.

A telephone conversation was scheduled with the investigator (midwife-perinatal psychology graduate) 15 days after hospitalization, in order to fill in the Impact of Event Scale Revised (IES-R) and Peritraumatic Dissociative Experiences Questionnaire (PDEQ). A second telephone conversation took place one month after the late fetal demise, to fill in the IES-R only. A third telephone conversation took place 3 months after the late fetal demise, again using only the IES-R.

The tools chosen were the IES-R and PDEQ scales. The IES-R (Impact of Event Scale-Revised) scale was validated in the study by Weiss et al.^19^, the French version was validated in 2003 by Brunet et al.^20^. This IES-R studies the semi-delayed and delayed consequences of a stressful event and is widely used in practice and in research as it is simple to use. This scale is used to detect patients at risk of having PTSD following a traumatic event. Early screening enables early intervention (such as counselling), thereby reducing the risk of long term PTSD^20–23^.

The IES-R is a 22-point questionnaire that measures the symptoms that define PTSD. The questions are in the form of propositions to which a patient responds according to possible answers graded from 0 to 4, ranging from "not at all" to "very much". The minimum score is 0 and the maximum score is 88. A score ≥ 33 correlates to a high number of PTSD symptoms and is widely accepted to be the optimal cut-off associated with a significant risk of PTSD^24^. Similarly, in our study, patients scoring ≥ 33 were considered to be at high risk of PTSD^25^.

Stress symptoms following a traumatic event are divided into two distinct diagnoses, each with a specific time frame. Symptoms that occur within 1 month of the trauma are primarily associated with acute stress whereas symptoms that appear after one month or that persist for more than a month is associated with PTSD. For this reason, patients were screened 1 and 3 months after late fetal demise. The primary end point of the study was the population at 1 month with an IES-R score ≥ 33.

The PDEQ (the Peritraumatic Dissociative Experiences Questionnaire) is a scale developed by Marmar et al.^19^ that assesses the dissociative symptoms at the time of a traumatic event (peritraumatic dissociation). This scale was validated in French in 2004 by Birmes et al.^24^. The PDEQ is a 10-point questionnaire in the form of propositions, the response methods are based on a Likert scale, each response is graded from 1 "not true at all” to 5 "extremely true". A score ≥ 15 demonstrates significant peritraumatic dissociation. In our study, we evaluated peritraumatic dissociation, it is described as an associated factor to symptoms of PTSD and it can easily be screened by caregivers^26,27^. Finally, we were also able to verify the link between peritraumatic dissociation and the development of PTSD symptoms in the event of late fetal demise.

In light of the prevalence of PTSD symptoms after late fetal demise, we expected to recruit a study population of 100 patients, during the 18 month inclusion period. Furthermore, it was decided to increase the population by 15% in order to compensate for patients lost during follow-up.

Results were reported as numbers and percentage for qualitative variables. Statistical analysis was made using Fisher's test and a Chi2 test. For quantitative variables, results were reported as a mean and a standard deviation. Statistical analysis was done a Mann–Whitney test or student-test. The significance threshold was set at 0.05 and all the tests were bilateral.

Changes in the symptoms of PTSD were assessed by calculating the differences in scores between each measurement pair. Study of the associated factors was done by univariate analysis. We looked for the factors linked to a score of ≥ 33 in the IES-R scale, at 1 and 3 months after late fetal demise.

## Results

**Figure 1:**
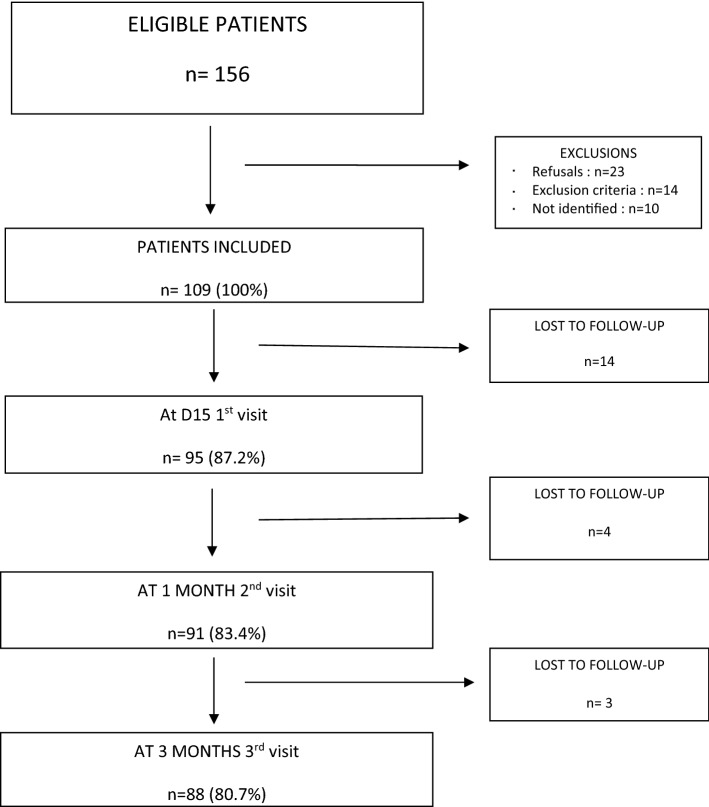
Flow chart.

A total of 156 patients experienced late fetal demise over the inclusion period and 109 patients (69.9%) were included in the study. Of the eligible patients, 23 declined to participate in the study, 14 were excluded and 10 were discharged without an offer to participate. Among the included patients, 88 patients were followed up until the end of the study. (cf. Fig. 1—Flow Chart).

Within the population corresponding to our primary endpoint, i.e. the population that responded to our telephone call at one month, the mean age of the patients included in the study was 31.7 (± 4.8) years, 87 patients (95.6%) were in a relationship and 76 (83.5%) patients had a job. 43 patients (47.3%) were nulliparous and 48 patients (52.7%) were multiparous.

Five patients (5.5%) had presented a history of psychiatric disorder (although not at the time of late fetal demise). Regarding gynaecologic history, 23 patients (25.3%) had experienced a spontaneous miscarriage, 12 (13.2%) had a history of voluntary termination of pregnancy (VTP), five patients (5.5%) had a history of ectopic pregnancy and finally, two patients (2.2%) had a history of LTP. (cf. Table 1).

**Table 1:** Population characteristics (n = 91) M1.

Characteristics	
Mean age (years), mean ± SD [min–max]	31.7 ± 4.8 [22–44]
**Marital status**	
In a relationship, number (%)	87 (95.6)
Cohabitation, number (%)	57(62.3)
Married, number (%)	24 (26.4)
Civil union, number (%)	6 (6.6)
Professional activity (yes), number (%)	76 (83.5)
Socio-professional category	
Employee, number (%)	32 (35.2)
Middle-level profession, number (%)	24 (26.4)
Worker, number (%)	11 (12.1)
Executive and higher intellectual profession, number (%)	5 (5.5)
Tradesman, dealer, business head, number (%)	4 (4.4)
**Gynaecological history**	
SM, number (%)	23 (25.3)
VTP, number (%)	12 (13.2)
EP, number (%)	5 (5.5)
LTP, number (%)	2 (2.2)
Nulliparous, number (%)	43 (47.3)
Multiparous, number (%)	48 (52.7)
Mean gestation, mean ± SD [min–max]	2.63 ± 1.9 [1–9]
**Psychiatric history**	
Depression, yes, number (%)	2 (2.2)
Anxiety disorders, yes, number (%)	2 (2.2)
EBD, yes, number (%)	1 (1.1)
**Addictions**	
Smoking, number (%)	13 (14.3)
Drugs, number (%)	0 (0)
Alcohol, number (%)	0 (0)

*%* percentage, *SD* Standard Deviation, *VTP* Voluntary termination of pregnancy, *LTP* Medical termination of pregnancy, *SM* Spontaneous miscarriage, *EP* ectopic pregnancy, *EBD* Eating Behaviour Disorder.

The mean term of late fetal demise was 23.7 WG ± 5.9. The earliest demise was 14 WG and the latest 40 WG. Late fetal demise was associated respectively with LTP among 59 patients (64.8%), FDIU for 25 patients (27.5%) and late miscarriage for 7 patients (7.7%).

Finally, 77 patients (84.6%) of the cohort chose to see their deceased infant in the delivery room and 51 (56.0%) chose to hold their dead infant in their arms. During hospitalization, within the department, 39 patients (42.9%) wished to see the body a second time. With regard to psychiatric treatment, 42 patients (46.1%) had a consultation with a psychologist or psychiatrist at their request during their stay at the hospital. (cf. Table 2).

**Table 2:** Contextual data on fetal demise (n = 91).

Data	
Mean term (WG) mean ± SD [min–max]	23.7 (± 5.9) [14–40]
**Features of pregnancy**	
Spontaneous pregnancy (yes), number (%)	78 (85.7)
Planned pregnancy (yes), number (%)	86 (94.5)
**Cause of fetal demise**	
Medical termination of pregnancy, number (%)	59 (64.8)
Fetal death in utero, number (%)	25 (27.5)
Late miscarriage, number (%)	7 (7.7)
**Reason late termination of pregnancy**	
Fetal reason, number (%)	55 (60.4)
Maternal reason, number (%)	4 (4.4)
**Features of childbirth**	
Spontaneous labour (yes), number (%)	15 (16.5)
Vaginal delivery (yes), number (%)	82 (90.1)
Fetus seen in delivery room (yes), number (%)	77 (84.6)
Fetus held by mother (yes), number (%)	51 (56.0)
**Post-partum**	
Fetus seen during hospitalization (yes), number (%)	39 (42.9)
Fetus declared to the civil registry (yes), number (%))	68 (74.7)
First name given (yes), number (%)	65 (71.4)
Funeral service by parents (yes), number (%)	19 (20.9)
Impressions for memory (yes), number (%)	65 (71.4)
Autopsy (yes), number (%)	44 (48.3)
Meeting with psychologist/psychiatrist during hospitalization (yes), number (%)	42 (46.1)
Psychological follow-up organized after hospitalization (yes), number (%)	13 (14.3)

*%* percentage, *SD* Standard Deviation, *WG* weeks of gestation.

With regard to the psychiatric scales, on the PDEQ, significant dissociation at the time of childbirth was identified among 83 patients (87.3%), and the mean score on the PDEQ was 23.8 (± 7.7) for a cut off ≥ 15. The mean score on the IES-R was 31.8 (± 12.5) 15 days after late fetal demise for a cut off ≥ 33, 25.0 (± 13.4) one month later and 17.6 (± 10.1) three months later. At 15 days following late fetal demise, 42 patients (44.2%) presented a pathological score (≥ 33) on the IES-R , at one month there were 31 patients (34.1%), and at three months there were 8 patients (9.1%) with a score ≥ 33. (cf. Table 3).

**Table 3:** Results on scales.

Data	
**D15**	
PDEQ score ≥ 15, number (%)	83 (87.3)
Mean PDEQ score, mean ± SD [min–max]	23.8 (± 7.7) [0–44]
IES-R score ≥ 33, number (%)	42 (44.2)
Mean IES-R score, mean ± SD [min–max]	31.8 (± 12.5) [4–63]
**M1**	
IES-R score ≥ 33, number (%)	31 (34.1)
Mean IES-R score, mean ± SD [min–max]	25.0 (± 13.4) [4–58]
**M3**	
IES-R scale ≥ 33, number (%)	8 (9.1)
Mean IES-R score, mean ± SD [min–max]	17.6 (± 10.1) [3–53]

*%* percentage, *SD* Standard Deviation, *IES-R* Impact of Event Scale-Revised, *PDEQ* Peritraumatic Dissociative Experiences Questionnaire.

After univariate analysis, factors associated with high IES-R scores were identified. Thirty one patients had a score ≥ 33 at one month after late fetal demise, or 34.1% of the total cohort at one month. These patients represented the population the most at risk in our study with a high number of symptoms of PTSD.

Women who had an episode of peritraumatic dissociation at the time of their delivery (PDEQ score > 15) had a significantly (100% *vs* 81.7%; p = 0.014) higher score on the IES-R. Although women who held the fetal corpse in their arms had a significantly higher IES-R score (71.0% *vs* 48.3%; p = 0.046). Patients who saw the corpse of their fetus a second time during their hospitalization also had a significantly higher scores on the IES-R (58.1% *vs* 35.0%; p = 0.035) as did those who organized their child’s funeral (32.3% *vs* 15.0%; p = 0.025). Term of pregnancy was not significantly related to high scores on the IES-R (p = 0.749), even though late fetal demise is not significantly associated with a high IES-R score, there is a trend towards a pathological IES-R score in case of late miscarriage (71.4% vs 28.6%; p = 0.052) (cf. Table 4).

**Table 4:** Associated factors/IES-R score ≥ 33 M1.

Data	IES-R < 33 (n = 60)	IES-R ≥ 33 (n = 31)	P value
Term ≤ 22 WG number (%)	25 (41.7)	14 (45.2)	0.749
Term > 22 WG, number (%)	35 (58.3)	17 (54.8)	
**Features of pregnancy**			
Spontaneous pregnancy (yes), number (%)	54 (90,0)	24 (77.4)	0.122
Induced pregnancy (assisted reproduction) (yes), number (%)	6 (10.0)	7 (22.6)	
Planned pregnancy (yes) number (%)	57 (95.0)	29 (93.5)	1.000
**Cause of fetal demise**			
Late termination of pregnancy, number (%)	43 (72.9)	16 (27.1)	0.052
Fetal death in utero, number (%)	15 (60.0)	10 (40.0)	
Late miscarriage, number (%)	2 (28.6)	5 (71.4)	
**Features of childbirth**			
Mental dissociative PDEQ ≥ 15	49 (81.7)	31 (100)	**0.014**
Fetus seen in delivery room (yes), number (%)	50 (83.3)	27 (87.1)	0.765
Fetus held (yes), number (%)	29 (48.3)	22 (71.0)	**0.046**
**Post-partum**			
Fetus seen during hospitalization (yes), number (%)	21 (35.0)	18 (58.1)	**0.035**
Fetus declared to the civil registry (yes), number (%))	43 (71.7)	25 (80.6)	0.448
Funeral service by parents (yes), number (%)	9 (15.0)	10 (32.3)	**0.025**

*%* percentage, *PDEQ* Peritraumatic Dissociative Experiences Questionnaire, *WG* weeks of gestation.

At 3 months we also found factors linked to the persistence of a high score on the IES-R: seeing the corpse of their fetus a second time during hospitalization (87.5% *vs* 12.5%; p = 0.019), the organization of funeral (62.5% *vs* 37.5%; p = 0.013) and also the existence of a pathological score at 1 month after fetal loss (87.5% *vs* 12.5%; p < 0.001).

However, peritraumatic dissociation is not connected to the persistence of a ≥ 33 score on the IES-R (p = 1.0) after 3 months.

## Discussion

The purpose of this study was to assess the prevalence of PTSD symptoms, according to DSM-5 criterion, in the short term following late fetal demise and, to identify the risk factors associated with PTSD.

15 days after late fetal demise 42 (44.2%) of the patients had a ≥ 33 score on the IES-R, which corresponds to a state of acute stress. Furthermore, in our study, more than one third of patients (34.1%) showed a pathological score (≥ 33) on the IES-R specific post-traumatic stress scale, at 1 month after late fetal demise. The mean score at one month after late fetal demise, within our population was 25.0 (± 13.4). This result is similar to that obtained by Kersting et al., in a meta-analysis of available literature published in 2012 wherein the average score on the IES-R at 3 months post late fetal demise was between 24.6 and 32.4^5^. This prevalence of PTSD symptoms shown in our study is significantly superior to the presence of PTSD symptoms which can be found in studies relating to delivery of a live child (3%)^8^.

In our study, the prevalence of symptoms of PTSD was lower than that in the study by Korenromp et al. which found a 39% prevalence for PTSD four months after LTP^12^, versus 34.1% in our population at one month and 9.1% at 3 months. This is explained by the fact that the cut-off score used on the IES-R in the study by Korenromp et al. was 26 and not ≥ 33 as is recommended in literature^29^. In another study by Jind^30^ of 56 patients, an 11% prevalence of pathological scores on a different screening scale was found four months after late fetal demise. These results are consistent with the mid-term prevalence identified in our population. Finally, Krosh^31^ reported a 17.6% prevalence of PTSD symptoms among 328 women, using the same scale (IES-R) and the same pathological threshold (≥ 33) as in our study. However, the time frame evaluation was not standardized as patients were recruited during their participation in discussion groups either several weeks or months after the late fetal demise.

Factors significantly associated with short-term PTSD symptoms among our population included: peritraumatic dissociation at the time of the late fetal demise (100% *vs* 81.7%; p = 0.014), holding the fetal corpse in one’s arms (71.0% *vs* 48.3%; p = 0.046), repeat exposure to the fetal corpse (58.1% *vs* 35%; p = 0.035), the organization of a funeral service (32.3% *vs* 15%; p = 0.025). Moreover, even though the significance threshold was not achieved in our study, there was a trend regarding PTSD symptoms and the reasons for late fetal demise; there is a trend towards a pathological IES-R score in case of late miscarriage (p = 0.052) (p = 0.052). In contrast, advanced term of pregnancy does not appear as a factor that significantly influences the onset of PTSD symptoms^4,28^. In our study, a term greater than 22 WG (threshold for viability according to French guidelines) was not found to be a component associated with an IES-R score ≥ 33 (p = 0.749).

A systematic review of literature published in 2017^4^, on PTSD in parents following infant death, the term of pregnancy at the time of fetal loss as well as the reason for fetal loss also did not appear to be significantly associated with the onset of intensity of PTSD. The author suggests that other associations are likely to influence the appearance of PTSD, such as the lack of support from family and friends.

With regard to the associated factors, a significantly higher rate of peritraumatic dissociation was found amongst women with a score ≥ 33 on the IES-R (p = 0.014). To our knowledge, peritraumatic dissociation at the time of late fetal demise has never been studied specifically, although it is a phenomenon described in traumatic circumstances^17,18^. In our study, peritraumatic dissociation was identified for a very large majority of women (87.3%) at the time of delivery.

Dissociative disorder is described as a detachment from oneself and/or one’s environment with difficulty in remembering information; it can appear at a moment of acute stress or during an event with high traumatic potential. In a meta-analysis of 53 studies^18^, 34 studies showed a significant relationship between peritraumatic dissociation at the time of a potentially traumatic event and the appearance of PTSD symptoms at a distance from the event. In our study, the high percentage (87.3%) of patients with a dissociative experience seems to confirm the fact that late fetal demise is a highly traumatic event. Peritraumatic dissociation, a risk factor to PTSD symptoms, is also associated with a high score on the IES-R in our cohort. Peritraumatic dissociation can also be easily tracked by obstetrical teams, using PDEQ during hospitalization, for example in order to refine monitoring and to better identify patients likely to develop PTSD symptoms.

In our study, patients who saw the fetal corpse a second time during their hospitalization had a significantly high score on the IES-R (p = 0.035). A few studies have looked at the impact on the mother of seeing her dead child and holding it in her arms. Redshaw et al.^15^, in a 2016 study including 468 women, found that women who held their dead baby in their arms had more symptoms than others (51.8% *vs* 36.5%). In our study, holding the fetal corpse in one’s arms also appears as a factor significantly associated with a score ≥ 33 on the IES-R (p = 0.046).

In our cohort, patients who chose to organize their child's funeral themselves also had a pathological scores on the IES-R (p = 0.025). This factor was included in a study^16^, of 65 women, relating to the persistence of PTSD symptoms (using the PTSD-1 interview scale) during future pregnancy following a late fetal demise. Organizing a funeral service (for 56% of the study population) was not found to be a factor significantly associated with PTSD (p = 0.14). However, although not always significant, these factors may provide an indication as to the intensity of the physical and emotional attachment.

In our study, for 8 women (9.1%), a score ≥ 33 on the IES-R was found to persist three months after late fetal demise. In fact, at 3 months follow up a score ≥ 33 on the IES-R is significantly correlated to a ≥ 33 score at 1 month (p < 0.001). Other factors associated with the persistence of PTSD symptoms include: repeat exposure to the fetal corpse during hospitalization (p = 0.019) and the organization of a funeral service by the parents (p = 0.013). Having a high IES-R score at 1 month appears to be strongly associated with the persistence of symptoms at 3 months after fetal loss. It also appears that the profile of patients with a score ≥ 33 at 3 months is similar to those with a score ≥ 33 at 1 month.

During hospitalization obstetrical teams should be aware of factors associated with PTSD or that indicate an intense physical and emotional attachment (holding the fetal corpse in one’s arms, the need to see the fetus during hospitalization and the organization of a funeral service) in order to offer patients considered at risk personalized support care. Societal perspectives concerning fetuses during late fetal loss have evolved over the last twenty years, the act of seeing or holding the corpse, declaring the deceased fetus to the civil registry or even organizing a funeral, showing an early emotional investment in the fetus. Caregivers must accompany women in these processes while being aware of the potential for trauma or, on the contrary, not impose these practices on patients.

The support of the healthcare team is of the highest importance during this time, and support for these women seems to be essential. Studies of PTSD after a live birth have shown that a poor rapport with caregivers at the time of delivery is a risk factor for PTSD^8,10^. It seems to us that the quality of this relationship is all the more important in the support provided by caregivers during late fetal loss. Other authors have highlighted the value of early intervention such as midwife-led counseling after a traumatic birth^21–23^. It appears that early intervention by a midwife may decrease the risk of developing PTSD. In our study, the persistence of a score ≥ 33 on the IES-R scale at 3 months post fetal loss was significantly associated with a score that was already pathological at one month (p < 0.001). This confirms the importance of screening patients as soon as possible in order to prevent PTSD symptoms from becoming chronic.The importance of social support by caregivers at the time of delivery also appeared as an essential factor in the mental outcome of women in the short term. Midwives are central to the management of these situations in addition to the care given to women and fetuses. Awareness and training are needed in order to improve the quality of relationships at the time of late fetal loss and to provide early supportive intervention. However, several meta-analyses have shown that early and systematic debriefing of a traumatic event can interfere with the spontaneous recovery process^32,33^. Our study provides insight into the identification of patients at risk of developing symptoms of PTSD. Early intervention by midwives should be proposed as a priority to these screened patients as well as, in a second phase, a more specific follow-up care, such as short cognitive therapies or Eye Movement Desensitization and Reprocessing (EMDR). These treatment strategies have shown to reduce the risk of developing PTSD after a traumatic birth^34,35^.

In spite of a sound methodology, our study does have some limitations, which include the unicentric nature of the study and the fact that the number of patients with a pathological score on the IES-R allowed only for a univariate analysis. Furthermore, as it is an exploratory study, no power calculations were done at the time of protocol. However, the univariate analysis did enable us to identify some significant associated factors that are coherent in comparison to literature. Furthermore, the measure of peritraumatic dissociation was done at 15 days after late fetal demise, a possible source of bias even if this timeframe is relatively short.

Moreover, we did not study the grieving process and especially complicated grief. As the loss of a child is associated with a grief experience that is particularly severe, long-lasting, and complicated, exploring grieving could be an interesting area of future research^36^. Also, there are PTSD risk factors which can be found in literature such as: exposure to previous trauma, complex psychopathology, social support, acute stress, the traumatic situation itself…^37^ Methodological strengths that enhance the internal validity of our results include the systematic follow-up of patients that enabled for an accurate short-term assessment of PTSD symptoms, the use of validated scales and the application of the reference cut-off score for detection of PTSD symptoms^29^.

## Conclusion

Late fetal demise is a significant risk factor for PTSD. Perinatal caregivers' awareness of trauma risk could improve the quality of their relationships with women during late fetal loss. Furthermore, other authors have been interested in the longer-term future of these women^4,38,39^, in particular during a subsequent pregnancy and even in the longer term. Early identification of patients at risk of developing PTSD symptoms is essential in order to adapt treatment, to reduce the risk of reactivation of the trauma and the long term persistence of PTSD symptoms. Obstetrical teams should track patient’s psychological status and remain vigilant of factors associated with PTSD or that indicate intense physical and emotional attachment. This screening would make it possible to offer these patients appropriate counseling by midwives at an early stage. Care could also be offered to spouses in order to improve the quality of support given to couples with a goal of reducing the traumatic impact of the event. It would be of value to supplement the results of this research with a secondary study carried out after adapted treatment strategies have been put in place to care for patients faced with a late fetal demise.
